# Association Between Total Testosterone Levels and Painful Temporomandibular Disorders in Women: A Pilot Case-Control Study

**DOI:** 10.7759/cureus.110460

**Published:** 2026-06-08

**Authors:** Shikha Yadav, Rajeev Aravindakshan, Vidya D Sripad, Jitendra Chawla

**Affiliations:** 1 Department of Dentistry, All India Institute of Medical Sciences (AIIMS), Mangalagiri, Guntur, IND; 2 Department of Community and Family Medicine, All India Institute of Medical Sciences (AIIMS), Mangalagiri, Guntur, IND; 3 Department of Biochemistry, All India Institute of Medical Sciences (AIIMS), Mangalagiri, Guntur, IND; 4 Department of Oral and Maxillofacial Surgery, All India Institute of Medical Sciences (AIIMS), Mangalagiri, Guntur, IND

**Keywords:** chronic joint pain, pain in women, temporo mandibular joint, temporomandibular pain, testosterone hormone, tmj disorders

## Abstract

Background: Temporomandibular disorders (TMDs) are prevalent musculoskeletal conditions affecting the temporomandibular joint (TMJ), masticatory muscles, and associated structures, causing significant orofacial pain and functional limitations. Epidemiological data show higher TMD prevalence in women, particularly of reproductive age, suggesting hormonal influences. While estrogen is well-studied, the role of testosterone remains unclear.

Objective: This pilot case-control study investigated the association between serum testosterone levels and painful TMDs in women aged 18-30 years.

Methods: Forty-seven female participants were recruited: 17 (36.2%) cases with clinically diagnosed painful TMDs and 30 (63.8%) age-matched controls without symptoms. Inclusion criteria included regular menstrual cycles (21-40 days); exclusion criteria included TMJ surgery or trauma, inflammatory joint diseases, hormonal disorders, and use of hormone-modifying medications. TMD diagnosis was based on the TMD pain screening questionnaire (scores ≥3 indicated cases). Serum total testosterone levels were measured using a Roche cobas® e 411 electrochemiluminescence immunoassay analyzer (Roche Diagnostics GmbH, Mannheim, Germany). Statistical analyses included analysis of covariance (ANCOVA) to compare testosterone levels and Pearson's correlation analysis to assess the relationship between testosterone levels and TMD duration.

Results: The mean serum testosterone level in the TMD group was 18.0 ng/dL (SD = 6.97), compared with 19.3 ng/dL (SD = 11.68) in the control group; the difference was not statistically significant (*P* = 0.90). No significant correlation was observed between testosterone levels and TMD duration or severity. Logistic regression indicated neither age nor testosterone significantly predicted TMD.

Conclusions: Serum testosterone levels are not significantly associated with the presence or severity of painful TMDs in women aged 18-30. These findings highlight the multifactorial nature of TMDs and suggest that testosterone may not play a primary role in their pathogenesis. Further research with larger samples and consideration of hormonal fluctuations during the menstrual cycle is warranted.

## Introduction

Temporomandibular disorders (TMDs) are complex musculoskeletal conditions affecting the temporomandibular joint (TMJ), masticatory muscles, and associated structures, significantly impacting an individual's quality of life [1]. These multifactorial disorders encompass a broad spectrum of clinical presentations, ranging from mild joint clicking and intermittent discomfort to debilitating chronic orofacial pain that interferes with daily activities such as chewing, speaking, and even sleeping. Common symptoms include masticatory muscle pain, TMJ discomfort, restricted jaw movement, and joint noise. The precise mechanisms underlying TMDs remain elusive, with research pointing to a confluence of factors, including anatomical variations, trauma, parafunctional habits, psychological stress, and hormonal influences [1]. Epidemiological studies indicate that TMDs affect approximately 34% of the global population, with regional variations: South America has the highest prevalence at 47%, followed by Asia (33%), Europe (29%), and North America (26%). The incidence peaks among individuals aged 18-60, particularly in women aged 20-40 years [2]. Studies have consistently demonstrated a higher prevalence of TMDs among women, particularly those of reproductive age, suggesting a potential link between female reproductive hormones and the development or exacerbation of these disorders [3]. Although circulating testosterone levels are lower in women compared to men, it remains the most abundant biologically active sex steroid in females and exerts significant physiological effects via widely distributed androgen receptors in musculoskeletal and neural tissues. Experimental evidence suggests a potential protective role of testosterone in TMJ nociception, which may partly explain the lower prevalence of TMD in males. Therefore, even within physiological ranges, variations in testosterone levels may influence pain modulation and TMD susceptibility in women. Exploring the relationship between testosterone levels and TMDs in women is imperative, given the hormone's role in modulating pain perception, muscle function, and inflammation. This study represents a pilot exploratory investigation into the association between painful TMDs and serum total testosterone levels in a female population. The primary objective was to evaluate the association between serum total testosterone levels and the presence of painful TMD by comparing affected individuals with healthy controls. A secondary objective was to assess whether the duration of TMD symptoms correlated with testosterone levels, thereby offering preliminary insights into chronicity and disease progression, both of which are implicated in the pathophysiology of TMD. As an exploratory pilot study, the findings are intended to generate preliminary data and effect-size estimates to inform future larger-scale investigations [4]. 

There is a lack of evidence in human females regarding the relationship between testosterone levels and painful TMDs. The null hypothesis posited no association between testosterone levels and painful TMDs.

## Materials and methods

Diagnosis and grouping

Painful TMDs were identified using the TMD Pain Screening Questionnaire, a validated instrument developed by Gonzalez et al. Participants scoring ≥3 were classified as cases, whereas those scoring 0-2 were categorized as controls [5]. Given the exploratory, pilot-study nature of the study and feasibility considerations, a standardized, screening-based diagnostic approach was employed to identify probable painful TMD among participants. All clinical examinations and participant assessments were performed by a single calibrated investigator to ensure consistency and minimize interobserver variability and assessment bias.

Data collection 

Demographic characteristics, menstrual history, and TMD symptom-related variables, including symptom onset, frequency, duration, menstrual phase association, diurnal variation, and perceived stress relation, were recorded using a structured proforma.

To evaluate symptom burden, a composite pain severity scale was developed using predefined symptom characteristics, including symptom duration (years), episode frequency, episode duration (days), temporal association with the menstrual cycle, diurnal variation, and stress association. Each component contributed to an aggregate severity score intended to reflect overall symptom chronicity and burden. To assess concurrent validity, the derived severity score was correlated with TMD screening questionnaire scores. The resulting moderate positive correlation (Pearson’s *r* = 0.522) supported its suitability for exploratory dose-response analyses within this pilot study.

Potential confounding variables were systematically documented. Body mass index (BMI) was calculated using measured height and weight (kg/m²). The menstrual phase at the time of assessment was recorded based on self-reported menstrual history, and perceived stress related to TMD symptoms was documented using a structured history and categorized according to participants' self-reports. These variables were included as covariates in adjusted statistical analyses to minimize confounding.

Blood sample collection and hormone analysis 

Following clinical evaluation, a trained phlebotomist collected 1 mL of venous blood from each participant into pre-labeled, coded vials to preserve blinding. Blood sampling was performed during routine outpatient visits according to a standardized collection protocol, and the menstrual phase at the time of sample collection was documented for all participants to account for potential hormonal fluctuations. Serum samples were analyzed for total testosterone concentration using a Roche cobas® e 411 electrochemiluminescence immunoassay analyzer (Roche Diagnostics GmbH, Mannheim, Germany), following standard laboratory operating procedures and manufacturer guidelines. Menstrual phase was subsequently incorporated as a covariate in adjusted analyses to minimize endocrine variability.

## Results

**Figure 1:**
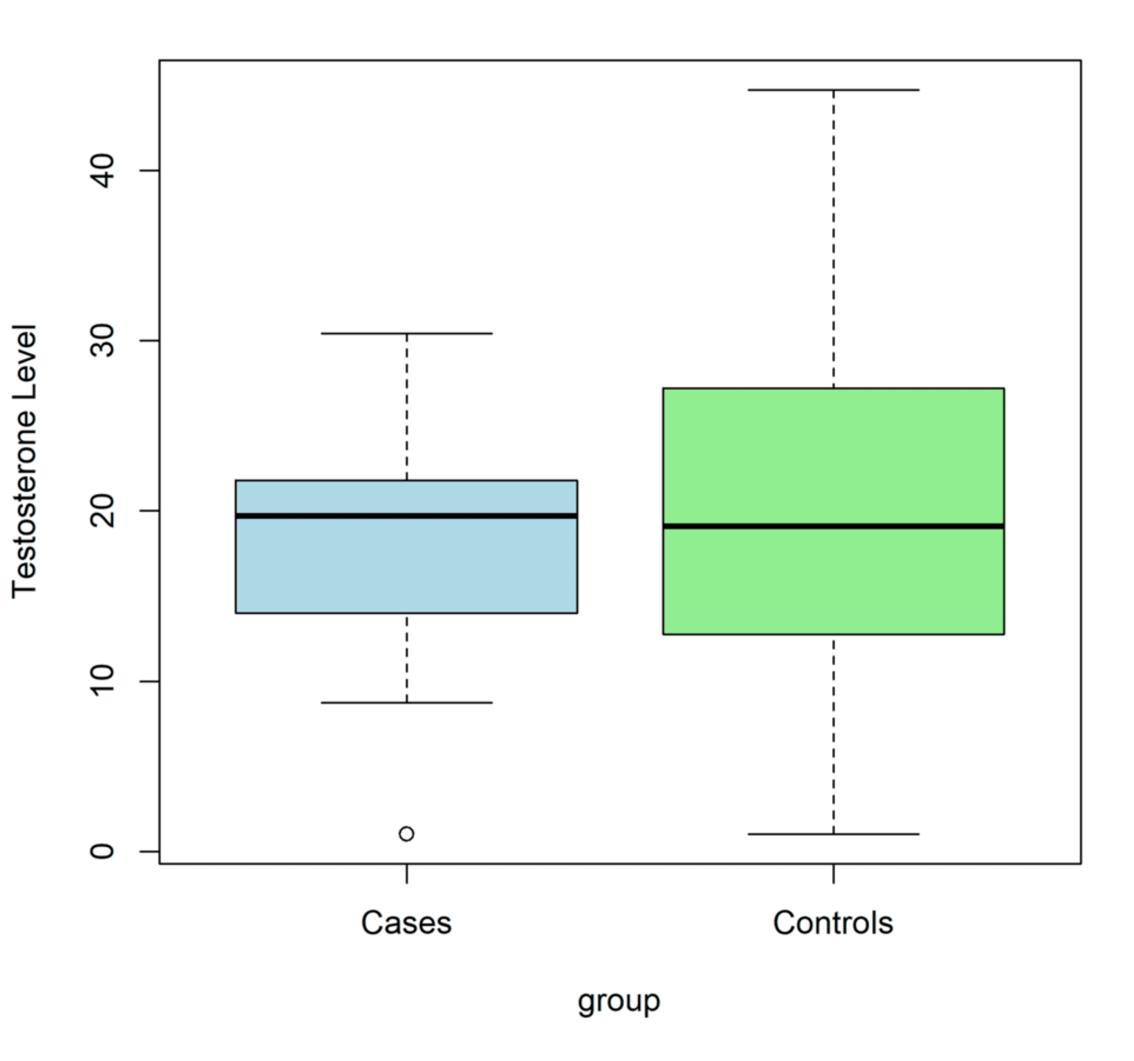
Distribution of testosterone levels in cases and controls. The box plot shows a broader distribution and greater variability of testosterone levels in controls compared to cases, with more extreme values in the control group.

This study conducted a comparative analysis between two distinct populations, specifically cases and controls, focusing on testosterone levels, while accounting for matched age distributions between groups. The case group, comprising 17 (36.2%) individuals, had an average age of 25.90 years with a standard deviation of 3.20. The control group consisted of 30 (63.8%) individuals with an average age of 23.93 years and a standard deviation of 3.47. Although the case group had a slightly lower mean age, there was no statistically significant difference in the age distribution between the two groups, as confirmed by an unpaired t-test. Testosterone levels in the case group were 18.0 ng/dL (SD = 6.97), compared with 19.3 ng/dL (SD = 11.68) in the control group. The graphical representation supports the lack of significance in the values between the two groups (p = 0.90) (Figure 1).

The study groups averaged approximately 20 ng/dL of testosterone (Figure 1). However, the distributions notably differed: the control group showed a broader spread and more outliers than the cases, and was tightly clustered around the median. 

**Figure 2:**
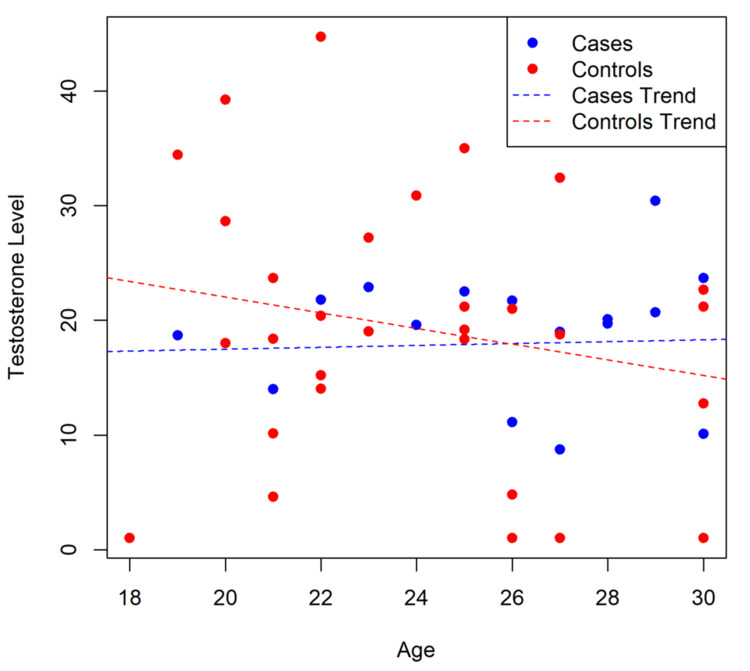
Relationship between age and testosterone levels in cases and controls. Testosterone levels are plotted against age for cases and controls, with trend lines showing a crossover at approximately 26 years.

The scatter plot of age and testosterone levels, with a smoothed trend line, showed a discontinuous crossover in testosterone levels between groups at approximately 26 years of age (Figure 2).

**Figure 3:**
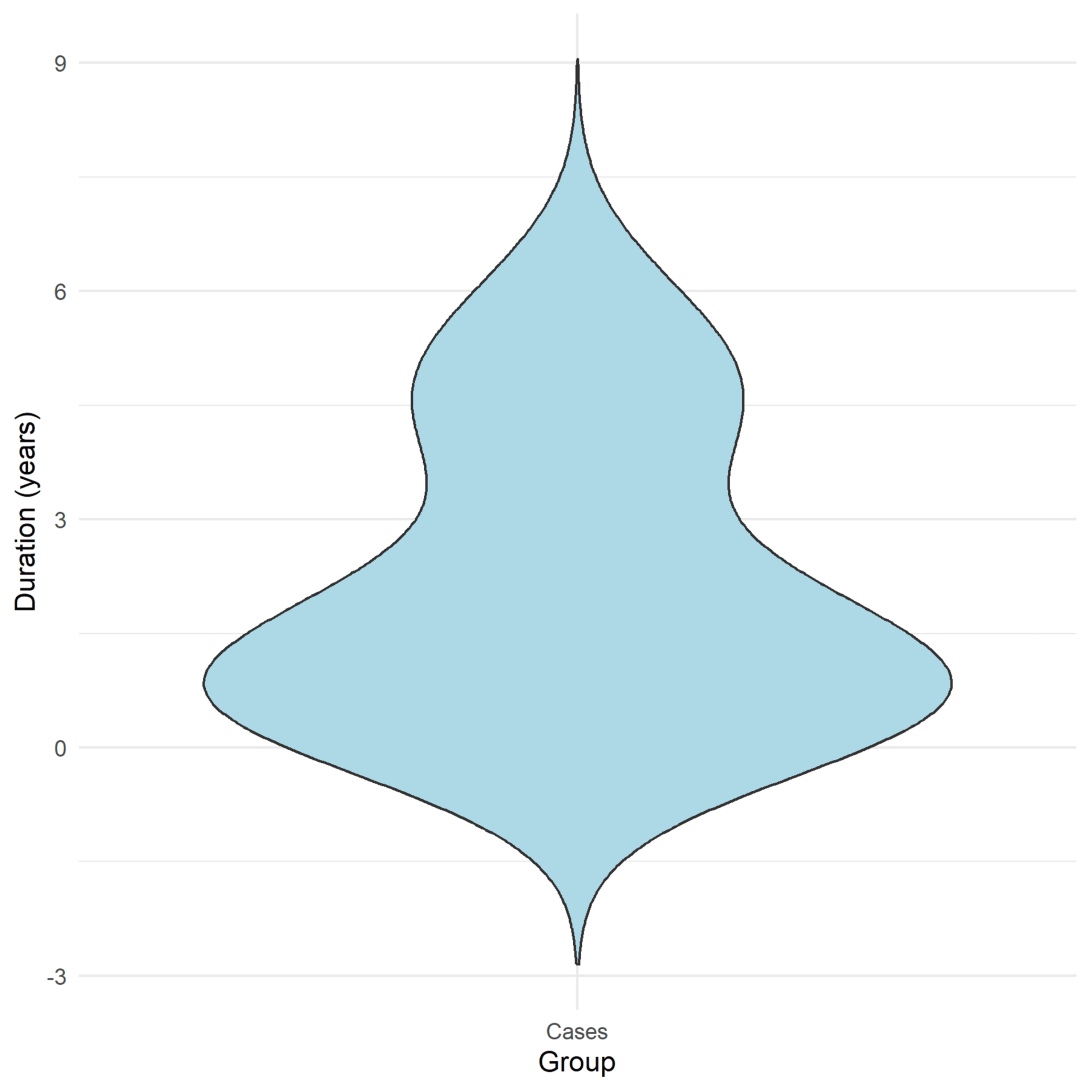
Distribution of condition duration among cases. The violin plot illustrates the distribution of condition duration among cases, showing a peak density at one to two years and a gradual decline toward longer durations.

In some cases, the overlaid density during the condition showed a peak of approximately one to two years. It tapers over the years (Figure 3). 

The logistic regression model improved case prediction when age and testosterone values were added to the equation, as indicated by a residual deviance of 57.89, compared to the null value of 61.51. The Akaike Information Criterion value of 63.89 suggested the same. The variables were not significant, but age confounded the model. In this study, neither age nor testosterone significantly explained the presence or the severity of pain, which captured the duration of pain and other attendant features of TMJ affection. The low values of the multiple R-squared (0.05683) and adjusted R-squared (−0.0779), along with the F-value of 0.4218 (*P* = 0.663), failed to support the hypothesis that testosterone is causally related to the origin of pain in TMJ complaints, even after adjusting for age as a confounder (Table 1).

**Table 1:** Multivariable logistic regression analysis of factors associated with painful temporomandibular disorders. Multivariable logistic regression analysis was performed to evaluate the association of age and total testosterone levels with painful temporomandibular disorders. Regression coefficients (β), standard errors, *z*-statistics, and *P*-values are reported. A *P*-value < 0.05 was considered statistically significant.

	Estimate	Standard error	*z*-value	Pr(>|*z*|)
Intercept	-4.780284	2.631806	-1.816	0.0693
Age	0.172528	0.097299	1.773	0.0762
Testosterone	-0.004652	0.032512	-0.143	0.8862

A total of 47 (100%) participants were included in the analysis, comprising 17 (36.2%) cases and 30 (63.8%) controls. The median age was 26.0 years (interquartile range (IQR) 24.0-28.0) in the case group and 23.5 years (IQR 21.0-26.0) in the control group, with no statistically significant difference (*P* = 0.059). Median testosterone levels were 20 ng/dL (IQR 14-22) for cases and 19 ng/dL (IQR 13-26) for controls, also showing no significant difference between groups (*P* = 0.90) (Table 2).

**Table 2:** Baseline characteristics and clinical features of cases and controls. Baseline characteristics and clinical features of cases and controls are summarized. Continuous variables are presented as median (IQR), and categorical variables as *n* (%). Group comparisons were conducted using the Wilcoxon rank-sum test and Fisher’s exact test, as appropriate. Statistical significance was defined as *P* < 0.05. Symptom-specific variables were available only for the case group. ^a^Median (IQR) or *n* (%). ^b^Wilcoxon rank-sum test or Fisher’s exact test. IQR, interquartile range

Variable	Case (*N* = 47, 100%), *n* = 17 (36.2%)^a^	Control (*N* = 47, 100%), *n* = 30 (63.8%)^b^	*P*-value
Age (years), median (IQR)	26.0 (24.0-28.0)	23.5 (21.0-26.0)	0.059
Testosterone (ng/dL), median (IQR)	20 (14-22)	19 (13-26)	0.9
Duration (years)	17 (100%)		>0.9
0.2	1 (5.9%)	0 (0%)	
0.5	6 (35%)	0 (0%)	
1	2 (12%)	0 (0%)	
2	3 (18%)	0 (0%)	
4	2 (12%)	0 (0%)	
5	2 (12%)	0 (0%)	
6	1 (5.9%)	0 (0%)	
Frequency	17(100%)		>0.9
1/month	1 (5.9%)	0 (0%)	
3/month	1 (5.9%)	0 (0%)	
4/month	1 (5.9%)	0 (0%)	
6/month	1 (5.9%)	0 (0%)	
10/month	1 (5.9%)	0 (0%)	
Continuous	12 (71%)	0 (0%)	
Duration_of_episode_days	8 (47.1%)		>0.9
0	1 (13%)	0 (0%)	
1	2 (25%)	0 (0%)	
2	1 (13%)	0 (0%)	
3	2 (25%)	0 (0%)	
5	2 (25%)	0 (0%)	
Specific_time_of_month	17 (100%)		>0.9
Nonspecific	15 (88%)	0 (0%)	
Pre-periods	1 (5.9%)	0 (0%)	
Periods and cold	1 (5.9%)	0 (0%)	
Specific_time_of_day	17 (100%)		>0.9
Morning	3 (18%)	0 (0%)	
Morning and night	1 (5.9%)	0 (0%)	
Night	2 (12%)	0 (0%)	
Nonspecific	11 (65%)	0 (0%)	
Remarks	6 (35.3%)		>0.9
Back-related issues	1 (17%)	0 (0%)	
Hard and chewy food, talking	1 (17%)	0 (0%)	
Stressed about pregnancy	2 (33%)	0 (0%)	
Stressed about family issues related to psychiatry	1 (17%)	0 (0%)	
When feeling stressed	1 (17%)	0 (0%)	

Among cases, the duration of the condition was most frequently reported as 0.5 years (6, 35%), followed by 2 years (3, 18%), 1 year (2, 12%), 4 years (2, 12%), 5 years (2, 12%), 6 years (1, 5.9%), and 0.2 years (1, 5.9%). Continuous pain was the most common frequency (12, 71%), with smaller proportions reporting 1, 3, 4, 6, or 10 episodes per month (1, 5.9% each). Most cases (11, 65%) reported no specific time of day for symptoms, while 3 (18%) noted symptoms in the morning, 2 (12%) at night, and 1 (5.9%) both in the morning and at night. The majority (15, 88%) had no relation to the menstrual cycle, while 1 (5.9%) reported symptoms pre-period and 1 (5.9%) reported symptoms during periods and in cold weather. Aggravating factors included stress (2, 33% pregnancy-related; 1, 17% family-related psychiatric; 1, 17% general), hard/chewy foods and talking (1, 17%), and back-related issues (1, 17%) (Table 2).

Logistic regression analysis using age and testosterone as predictors showed neither variable to be statistically significant (age: β = 0.1725, standard error (SE) = 0.0973, *P* = 0.0762; testosterone: β = -0.0047, SE = 0.0325, *P* = 0.8862). Adding both predictors reduced the residual deviance from 61.51 (null model) to 57.89, with an Akaike Information Criterion (AIC) of 63.89, suggesting a modest improvement in model fit. Age appeared to exert a mild confounding effect. However, model fit remained poor (multiple *R*² = 0.0568; adjusted *R*² = -0.0779; *F* = 0.4218; *P* = 0.663), indicating that neither age nor testosterone explained the presence or severity of pain (Table 1).

Testosterone levels did not differ significantly between cases and controls, and neither age nor testosterone predicted TMJ-related pain in this pilot study.

## Discussion

TMDs encompass a spectrum of musculoskeletal and neuromuscular conditions that affect the TMJ, masticatory muscles, and associated structures. Clinically, TMDs manifest as pain in the jaw, face, or neck, joint sounds such as clicking or crepitus, and limitations in mandibular movement, which can significantly impair quality of life [6].​

The etiology of TMDs is multifactorial and involves a complex interplay of genetic, hormonal, environmental, and psychological factors (Table 2). Despite extensive research, the precise etiology of TMDs remains multifactorial and not fully understood. Factors such as occlusal discrepancies, parafunctional habits, psychological stress, and hormonal influences have been identified as contributory [7]. Recent advances have highlighted the significance of the *COMT *gene, with specific genetic variants (haplotypes) associated with an increased risk of TMD development and enhanced pain sensitivity [8]. Diagnostic criteria for TMD require simple, straightforward, reliable, and valid operational definitions for the history, examination, and imaging procedures to facilitate physical diagnoses in both clinical and research settings [9]. 

It is well established that women are disproportionately affected by TMDs, with prevalence rates exceeding those in men by a factor of 1.5 to 2.11. Several hypotheses have been proposed to explain this gender disparity, including differences in pain perception, hormonal influences, and psychosocial factors. Specifically, fluctuations in estrogen and progesterone levels during the menstrual cycle, pregnancy, and menopause have been implicated in the pathogenesis of TMDs in women. Estrogen, in particular, is involved due to its role in modulating pain sensitivity and joint function. Studies have shown that fluctuations in estrogen levels, such as those occurring during the menstrual cycle or with hormone replacement therapy, may influence TMD symptoms (Table 2) [10,11]. 

However, less attention has been given to the potential role of testosterone, an androgenic hormone, in lower concentrations in women than in men. However, it still possesses significant physiological effects through androgen receptors distributed throughout the body, including the temporomandibular joint and surrounding tissues. Appropriate testosterone levels are vital for both men's and women's physical, mental, and emotional health [7]. Testosterone, traditionally viewed as the primary male sex hormone, plays a multifaceted role in female physiology, influencing muscle mass, bone density, mood regulation, and pain perception [7,12]. Glaser and Dimitrakakis suggest that testosterone may protect against TMDs [7]. The lower prevalence of TMD among males compared to females may be attributed to testosterone's potential protective effect against TMJ nociception [13]. Animal studies indicate that testosterone can mitigate temporomandibular joint damage, particularly in the presence of inflammation [7]. The present case-control study did not find a significant association between total testosterone levels and painful TMDs in women, implying that testosterone levels alone may not be a primary determinant in the pathophysiology of TMD pain. Animal experiments have demonstrated that this protective effect of testosterone against TMJ nociception is mediated through androgen receptors [14].

Cycle-phase fluctuations in female serum testosterone are statistically significant, necessitating consideration of menstrual timing in the interpretation of diagnostic assays and research measurements; mid-cycle values consistently exceed concentrations observed during menses or the early follicular phase. Although the amplitude of testosterone variation is less pronounced than that of estradiol or progesterone surges during ovulation, these changes are nonetheless sufficient to mediate physiologically relevant effects on biochemical, behavioral, and reproductive pathways in women of reproductive age.

While a definitive causal relationship between testosterone and TMD remains unclear, exploring the potential modulatory effects of testosterone on pain pathways, muscle physiology, and inflammatory responses within the TMJ may provide valuable insights into the underlying mechanisms of TMD, and investigating the hormonal influence on TMDs, with a focus on testosterone, may lead to the development of more targeted and effective treatment strategies [7,13]. There is no ideal pain therapy for TMD, and treatment strategies must be tailored to each patient and their specific condition [15]. Given the heterogeneity of TMDs and the complexity of their underlying mechanisms, emphasis should be placed on a holistic approach and individualized strategy for treating TMD [16-18].

The null hypothesis that no association exists between testosterone levels and painful TMD was accepted.

Strengths and limitations

This study has several strengths. It represents one of the few exploratory investigations examining the association between serum total testosterone levels and painful TMDs in young adult women, an underexplored area with potential clinical and biological significance. The prospective case-control design, clearly defined inclusion and exclusion criteria, and adjustment for relevant covariates such as age, BMI, stress, and menstrual phase enhanced internal validity. Furthermore, the use of a standardized chemiluminescence immunoassay platform for serum testosterone estimation and the application of appropriate statistical methods strengthened methodological rigor.

However, several limitations should be acknowledged. First, the small sample size and pilot nature of the study may have limited statistical power, increasing the possibility of type II error and restricting the generalizability of findings. Second, although the menstrual phase at the time of assessment and blood collection was documented and adjusted for during analysis, hormonal sampling was not standardized to a specific phase of the menstrual cycle, and residual endocrine variability may have influenced testosterone measurements. Third, only total testosterone levels were assessed; additional hormonal markers, including free testosterone, sex hormone-binding globulin (SHBG), estradiol, and related endocrine parameters, were not evaluated, limiting biological interpretation. Fourth, painful TMD was identified using a validated screening questionnaire rather than a comprehensive clinical diagnostic protocol such as the Diagnostic Criteria for Temporomandibular Disorders (DC/TMD), which may affect diagnostic precision. Finally, although important confounders such as BMI, menstrual phase, and perceived stress were considered, a more comprehensive assessment using validated psychometric and hormonal measures may improve future investigations.

Future studies with larger, adequately powered cohorts, standardized hormonal sampling across menstrual phases, and comprehensive endocrine and clinical diagnostic assessment are warranted to better elucidate the role of testosterone in painful TMD. Further research may also help determine whether hormonal modulation, including testosterone-related mechanisms, has therapeutic implications in chronic TMD pain conditions.

## Conclusions

In conclusion, this pilot case-control study did not identify a statistically significant difference in serum total testosterone levels between women with painful TMDs and healthy controls under the conditions of this study. However, given the exploratory nature of the study, limited sample size, and potential hormonal variability, these findings should be interpreted with caution and should not be considered evidence of the absence of an association. The multifactorial nature of TMDs, involving complex interactions among hormonal, biomechanical, psychosocial, and inflammatory factors, underscores the need for further adequately powered studies with standardized hormonal assessment and comprehensive endocrine evaluation to better elucidate the potential role of testosterone in TMD pathophysiology and its possible therapeutic implications.
